# Child Behavioral Scores Correlate With Prenatal Tobacco and Marijuana Exposure, Sociodemographic Variables and Interactions of Default Mode and Dorsal Attention Networks

**DOI:** 10.1002/brb3.71168

**Published:** 2026-01-15

**Authors:** Ramana V. Vishnubhotla, Yi Zhao, Rupa Radhakrishnan

**Affiliations:** ^1^ Department of Radiology and Imaging Sciences Indiana University School of Medicine Indianapolis Indiana USA; ^2^ Department of Biostatistics and Health Data Science Indiana University School of Medicine Indianapolis Indiana USA

**Keywords:** ABCD, attention networks, prenatal marijuana exposure, prenatal tobacco exposure

## Abstract

**Introduction:**

Prenatal substance exposure is an increasing problem that has been linked to multiple neurodevelopmental impairments and alterations to brain functional connectivity.

**Methods:**

Behavioral scores and functional network correlation data were obtained from the Adolescent Brain Cognitive Development^SM^ (ABCD) Study. First, behavioral scores based on the child behavioral checklist were tested for associations with prenatal exposure to several substances along with demographic data. Then differences in resting‐state functional networks were assessed based on prenatal substance exposure. Third, we assessed the impact of resting‐state functional networks on behavioral scores. A linear regression was used for all these analyses, and a false discovery rate < 0.05 was considered significant.

**Results:**

Based on the selection criteria, 6674 subjects were included in the analysis. Prenatal tobacco exposure (PTE), prenatal marijuana exposure, household income, and food insecurity were associated with worse behavioral scores. Additionally, PTE was significantly associated with increased connectivity between the default mode network (DMN) and dorsal attention network (DAN) and decreased intra‐network connectivity within the DAN. Finally, there were five CBCL scales that were associated with differences in network connectivity.

**Conclusion:**

Taken together, these results suggest PTE to be associated with multiple functional networks, including those associated with several CBCL scales.

## Graphical abstract

This secondary analysis of data from 6674 subjects from the Adolescent Brain Cognitive DevelopmentSM (ABCD) repository revealed prenatal tobacco exposure (PTE), prenatal marijuana exposure, household income and food insecurity to be associated wtih worse scores on the child behavioral checklist (CBCL). PTE was significantly associated with increased connectivity between the default mode network (DMN) and dorsal attention network (DAN) and decreased intra‐network connectivity within the DAN. There were five CBCL scales that were associated with differences in network connectivity.

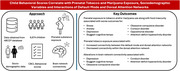

**Abbreviations**
ABCD: Adolescent brain cognitive development^SM^
ADHD: Attention deficit and hyperactivity disorder
CBCL: Child behavioral checklist
DAN: Dorsal attention network
DMN: Default mode network
DSM: Diagnostic and statistical manual of mental disorders
FDR: False discovery rate
fMRI: Functional magnetic resonance imaging
MRI: Magnetic resonance imaging
NDA: NIMH data archive
NIMH: National institute of mental health
PME: Prenatal marijuana exposure
PTE: Prenatal tobacco exposure
ROI: Region of interest
VAN: Ventral attention network

## 1 Introduction

Substance use during pregnancy is a major healthcare concern. Tobacco, alcohol, and marijuana are the most frequently used substances in pregnancy, followed by cocaine and opioids (Prince et al. 2023). According to the National Center for Health Statistics (Drake et al. 2018), 7.2% of women smoked tobacco during pregnancy, with the highest prevalence (10.7%) observed among those aged 20 to 24 years (Drake et al. 2018). The Center for Disease Control and Prevention reports 13.5% of women drinking while pregnant, with binge drinking in 5.2% of them (Gosdin et al. 2022). Marijuana use during pregnancy has a reported prevalence of 10% in pregnant women aged 18 to 25 years (Young‐Wolff et al. 2017). However, these numbers may underestimate actual usage, as 19% of pregnant women aged 18 to 25 years tested positive for marijuana in urine screens. Moreover, the COVID‐19 pandemic has worsened substance use in pregnancy (Young‐Wolff et al. 2021).

Prenatal exposure to alcohol, tobacco, and illicit substances is associated with adverse neurodevelopmental outcomes, including an increased risk of developmental disorders such as attention deficit hyperactivity disorder (ADHD) (Garrison‐Desany et al. 2022; Scott‐Goodwin et al. 2016). Prenatal tobacco exposure (PTE) and prenatal marijuana exposure (PME) have been linked to poor autonomic regulation during development (Eiden et al. 2018). Studies show that PME is associated with developmental deficits, including gaps in problem‐solving skills and memory, increased depressive and anxiety symptoms, and decreased attentiveness in school‐aged children (de Moraes Barros et al. 2008; Goldschmidt et al. 2000; Leech et al. 2006; Thompson et al. 2019). Based on a meta‐analysis by He et al., PTE is associated with an increased risk of ADHD in children (2020). Another study showed that alcohol use combined with exposure to environmental tobacco smoke increased the risk of ADHD (Han et al. 2015). Prenatal exposure to other substances, such as marijuana (Roncero et al. 2020), cocaine (Morrow et al. 2009), and opioids (Tronnes et al. 2021) is also shown to increase the likelihood of developing ADHD.

Prenatal exposure to substances also impacts brain network development and connectivity that persists to adolescence that may be assessed through functional MRI (fMRI) (Faraj et al. 2023; Radhakrishnan et al. 2022; Radhakrishnan et al. 2022; Sundermann et al. 2023; Thomason et al. 2021). For example, PME may exhibit decreased response inhibition and altered brain activation when performing executive functioning tasks (Smith et al. 2016). Altered brain activation is shown to be associated with poorer inhibitory control on magnetic resonance imaging (fMRI) in adolescents with prenatal substance exposure (Roos et al. 2017). Brain functional networks are also shown to be impacted in conditions with other developmental and behavioral disorders such as ADHD (Wang et al. 2020; Zhang et al. 2020).

Using resting state functional MRI (rs‐fMRI), recent alterations in intra‐ and inter‐network connections have been shown with substance addiction (Buckner and Vincent 2007; Grodin et al. 2017; Liang et al. 2015; Peterson et al. 2014; Sutherland et al. 2012; Zhang and Volkow 2019). In adults with substance exposure, these networks typically impacted include the default mode network (DMN) (Liang et al. 2015), the executive function network (Cole and Schneider 2007), the salience network (SN) and the attention networks (Menon and Uddin 2010; Seeley et al. 2007). Alterations in intra‐ and internetwork brain functional connectivity are also shown in infants with prenatal opioid exposure, where there is a preferentially greater impact on inter‐ rather than intra‐network brain functional connectives (Jiang et al. 2022). Altered intra‐network and inter‐network brain functional connectivity is also seen with developmental disorders such as autism spectrum disorder. A prior small‐scale study suggested that increased intra‐network and reduced inter‐network functional connectivity seen in adolescents with prenatal alcohol exposure may be due to inefficient network specialization and impaired long‐range functional connectivity within the attention network regions in these individuals.

Given the complex nature of neurodevelopment, it is challenging to isolate developmental deficiencies to a single factor—that is, prenatal substance exposure alone. The biopsychosocial model is a methodology to account for the interplay of multiple factors, including the biological, psychological, and societal (Borrell‐Carrio et al. 2004; Engel 1977), that impact childhood development. A biopsychosocial model may provide a plausible role for the various factors that may influence impaired development in prenatal substance exposure and allow for the creation of more personalized and effective treatment strategies. This study will leverage the existing large Adolescent Brain Cognitive Development^SM^ (ABCD) cohort to test associations between multiple substances prenatally, sociodemographic factors, and adolescent behavioral outcomes along with underpinning alterations in intra‐ and inter‐network brain functional connectivity.

## 2 Methods

### 2.1 Gathering Data

Data was acquired from the Adolescent Brain Cognitive Development^SM^ (ABCD) Study 4.0 (https://abcdstudy.org), held in the NIMH Data Archive (NDA) (Alcohol Research: Current Reviews Editorial Staff, 2018; Casey et al. 2018). Data extracted was from baseline visits and included sociodemographic data such as age, sex, race, birth weight, maternal education, food insecurity, annual household income, and any prenatal substance exposure. Combined household annual income was categorized into fewer bins for easier assessment. Housing insecurity was also assessed but had a high correlation with food insecurity and was excluded to prevent multicollinearity. Functional neuroimaging resting‐state network correlation data and parent‐reported child behavioral checklist (CBCL) (Achenbach and Ruffle 2000) scores were also obtained from the database. Inclusion criteria included the presence of functional resting‐state network data, framewise displacement less than 0.3 mm, and scans performed at the baseline. Subjects with missing dates in critical demographic variables, such as age, sex, substance exposure, food insecurity, income data, or CBCL metrics, were excluded. A final cohort of 6674 subjects was used for this study.

### 2.2 Behavioral Metrics

Behavioral metrics based on a parent‐reported child behavioral checklist (CBCL) (Achenbach and Ruffle 2000) were used. Standardized summarized scale scores based on six Diagnostic and Statistical Manual of Mental Disorders, Fourth Edition (DSM‐IV) scales (Nakamura et al. 2009) (Depression, Anxiety Disorder, Somatic Problems, ADHD, Oppositional Disorder and Conduct Disorder) and three CBCL 2007 scale scores (Sluggish Cognitive Tempo (SCT) Obsessive‐Compulsive Problems (OCD), and Stress Problems) were included (Achenbach and Rescorla 2001).

### 2.3 Resting‐State Functional MRI Networks

Functional MRI resting‐state network data was obtained via the NDA as described in the Gathering Data section. Resting state functional MRI correlation values between networks were used. Resting‐state network data that were included for analysis were based on prior studies in substance use disorder and neurodevelopment and included the default mode, salience, dorsal attention, ventral attention, auditory, and visual networks.

### 2.4 Biopsychosocial Model

Factors were categorized as biological, psychological, or societal (Borrell‐Carrio et al. 2004; Engel 1977). Biological factors included age, sex, birth weight, prenatal substance exposure, and brain network connectivity. Psychological factors included the different subsets of the Childhood Behavior Checklist (CBCL). Societal factors included household income, maternal education, food insecurity, and race.

### 2.5 Statistical Analysis

Statistical analyses were performed using Microsoft Excel (https://office.microsoft.com/excel; Microsoft Corporation; Redmond, WA), R Project for statistical computing version 4.3.2 (https://www.r‐project.org/), MATLAB (mathworks.com/products/matlab.html; Mathworks; Natick, MA), and within individual processing packages.

### 2.6 Association of Prenatal Substance Exposure on Behavioral Scores

Correlations of demographic characteristics and prenatal substance exposure were assessed with linear regression. Demographic factors such as age, sex, birth weight, maternal education, food insecurity, household income, and race were used as predictor variables. Since food insecurity was highly correlated with housing insecurity, only food insecurity was used in the linear regression. Additionally, prenatal exposure to tobacco, alcohol, marijuana, cocaine, and opioids was also modeled as predictor variables. Summarized behavioral scales including stress problems, anxiety disorder, depression, somatic problems, SCT, OCD, conduct disorder, oppositional defiant disorder, and ADHD scores were used as the response variables. Multiplicity was corrected using the Benjamini‐Hochberg procedure to control for the false discovery rate (FDR) (Benjamini and Hochberg 1995). An FDR of < 0.05 was considered significant.

### 2.7 Association of Prenatal Substance Exposure on Network Connectivity

A Fisher's transformation was applied to the between resting‐state network correlation data. A linear regression was performed to assess the impact of demographic characteristics and prenatal substance use on between‐network connectivity. Predictor variables include demographic variables such as age, sex, birth weight, maternal education, food insecurity, household income, and race and prenatal substance exposure variables such as exposure to tobacco, alcohol, marijuana, cocaine, and opioids. Scanner manufacturer information was also included to account for site‐based differences. Between‐network Fisher's transformed correlation data provided was used as the response variable. Multiplicity was corrected using the Benjamini‐Hochberg procedure to control for the FDR (Benjamini and Hochberg 1995). An FDR of < 0.05 was considered significant.

### 2.8 Association of Network Connectivity With Behavioral Scores

A Fisher's transformation was applied to the between resting‐state network correlation data. A linear regression was performed to assess the impact of demographic characteristics and prenatal substance use on between‐network connectivity. Only demographic and substance exposure predictor variables significant in the prior two analyses were included. Scanner manufacturer information and between‐network Fisher's transformed correlation values were also included as predictor variables. Significance of network correlation associations was assessed with respect to each behavioral scale. Multiplicity was corrected using the Benjamini‐Hochberg procedure to control for the FDR (Benjamini and Hochberg 1995). An FDR of < 0.05 was considered significant.

### 2.9 Association of Network Connectivity With Behavioral Scores in Subgroups

The groups were divided into multiple categories involving exposure to individual substances (those that were significant in prior analyses) and the combination of these exposures. Then, a linear regression was performed to assess the impact of demographic characteristics and prenatal substance use on between‐network connectivity. Only demographic and substance exposure predictor variables significant in the prior two analyses were included. Significance of network correlation associations was assessed with respect to each behavioral scale for each subgroup. Multiplicity was corrected using the Benjamini‐Hochberg procedure to control for the FDR (Benjamini and Hochberg 1995). An FDR of < 0.05 was considered significant.

## 3 Results

### 3.1 Demographics

A total of 6674 subjects were included (3240 male) with an age range of 9 and 11 years. Prenatal exposure to tobacco was the most frequent, and prenatal exposure to cocaine was the least common amongst these subjects. 3563 mothers had a college degree, and 3111 mothers had no college degree. Data is summarized in Table 1.

**TABLE 1:** Demographic data.

Total subjects	6674
Sex	
Male	3240
Female	3434
Baseline age in months (SD)	119.6 (7.5)
Birth weight in lbs. (SD)	7.03 (1.5)
Prenatal substance exposure	
Tobacco	262
Alcohol	180
Marijuana	105
Cocaine	7
Opioid	9
Food insecurity	469
Maternal education	
Had a college degree	3563
No college degree	3111
Race	
White	5176
Black/African American	829
Native American/Pacific Islander	248
Asian	127
Other/mixed	294
Household income (stratified)	
Below $50,000	1740
Between $50k to $100k	1906
Between $100k to $200k	2172
Above $200,000	856

### 3.2 Prenatal Exposure to Tobacco and Marijuana Associated With Poorer CBCL Scores

A single linear regression was performed to assess the significance of biological characteristics and prenatal substance exposure on reported CBCL scores. PTE was significantly associated with all scales, and PME was significantly associated with 7 out of 9 scales (Table 2). Prenatal exposure to alcohol, cocaine, or opioids was not associated with behavioral scores. When assessing demographics, sex and food insecurity were associated with all scales. Boys had poorer scores on eight of the scales, while girls had poorer scores for somatic problems (Table 2). Age was not associated with behavioral scores, and birth weight was only positively associated with OCD but no other scores.

**TABLE 2:** The association of prenatal substance exposure and sex with behavioral scores was tested using a general linear model (*n* = 6,674).

	**Tobacco**		**Marijuana**		**Sex**	
	***t*‐stat**	***p*‐FDR**	***t*‐stat**	***p*‐FDR**	***t*‐stat**	***p*‐FDR**
Stress	3.44	0.001*	5.08	< 0.001*	5.76	< 0.001*
Anxiety	2.05	0.041*	3.13	0.003*	4.24	< 0.001*
Depression	2.93	0.003*	1.90	0.064	3.48	< 0.001*
Somatic	2.44	0.015*	0.32	0.751	−3.33	< 0.001*
Sluggish	2.13	0.033*	4.35	< 0.001*	3.52	< 0.001*
OCD	2.05	0.040*	2.88	0.005*	4.05	< 0.001*
Conduct	6.31	< 0.001*	5.49	< 0.001*	3.94	< 0.001*
Oppositional	5.15	< 0.001*	4.25	< 0.001*	6.72	< 0.001*
ADHD	3.22	0.001*	5.15	< 0.001*	5.48	< 0.001*

*Denotes significance.

A false discovery rate (FDR) < 0.05 was considered significant.

Abbreviations: ADHD, attention deficit hyperactivity disorder; OCD, obsessive compulsive disorder.

### 3.3 Societal Factors Associated With CBCL Scores

In the same analysis, we assessed the association of sociological variables such as household income, food insecurity, maternal education, and race on CBCL scores. Not surprisingly, higher annual household incomes were associated with better behavioral scores in all 9 scales (Table 3). Additionally, food insecurity was linked with poorer behavioral outcomes for all scales. Being Black/African American was significantly associated with lower scores (better outcomes) in 7 out of 9 scales than White adolescents (Table 4).

**TABLE 3:** The correlation of income levels (compared to annual household income <$50K) or food insecurity and behavioral scores was assessed using a general linear model (*n* = 6674).

	$50k to $100k		$100k to $200k		> $200k		Food insecurity	
	*t*‐stat	FDR	*t*‐stat	FDR	*t*‐stat	FDR	*t*‐stat	FDR
Stress	−4.10	< 0.001*	−6.18	< 0.001*	−7.92	< 0.001*	9.08	< 0.001*
Anxiety	−2.69	0.011*	−4.37	< 0.001*	−6.82	< 0.001*	6.14	< 0.001*
Depression	−3.87	< 0.001*	−6.24	< 0.001*	−7.08	< 0.001*	10.45	< 0.001*
Somatic	−0.60	0.583	−3.08	0.002*	−4.72	< 0.001*	6.82	< 0.001*
Sluggish	−2.75	0.011*	−3.76	2.23E‐04*	−4.75	< 0.001*	6.21	< 0.001*
OCD	−0.55	0.583	−2.00	0.045*	−4.47	< 0.001*	7.29	< 0.001*
Conduct	−5.46	< 0.001*	−6.79	< 0.001*	−6.67	< 0.001*	8.11	< 0.001*
Oppositional	−3.13	0.004*	−4.33	< 0.001*	−5.53	< 0.001*	7.93	< 0.001*
ADHD	−2.59	0.012*	−4.36	< 0.001*	−5.73	< 0.001**	7.90	< 0.001*

*Denotes significance.

A false discovery rate (FDR) < 0.05 was considered significant.

**Abbreviations**: ADHD, Attention deficit hyperactivity disorder; OCD, Obsessive compulsive disorder.

**TABLE 4:** The correlation of race (compared to those who are White) and behavioral scores was assessed using a general linear model (*n* = 6674).

	African American		Native American		Asian		Other/Mixed	
	*t*‐stat	FDR	*t*‐stat	FDR	*t*‐stat	FDR	*t*‐stat	FDR
Stress	−4.00	< 0.001*	0.09	0.926	−0.90	0.635	−1.96	0.110
Anxiety	−6.32	< 0.001*	−0.74	0.829	−1.41	0.400	−1.51	0.147
Depression	−5.52	< 0.001*	−1.13	0.625	0.27	0.790	−2.53	0.051
Somatic	−3.48	< 0.001*	0.40	0.857	−1.35	0.400	−0.66	0.510
Sluggish	−1.73	0.095	2.75	0.054	−1.49	0.400	−1.87	0.110
OCD	−5.17	< 0.001*	−0.54	0.857	−0.63	0.676	−2.22	0.080
Conduct	−5.46	< 0.001*	1.09	0.625	−0.27	0.790	−1.64	0.130
Oppositional	−3.13	0.002*	−0.30	0.857	−2.00	0.400	−2.89	0.035*
ADHD	0.55	0.584	1.80	0.326	−0.80	0.635	−1.69	0.130

* Denotes significance.

A false discovery rate (FDR) < 0.05 was considered significant.

**Abbreviations**: ADHD, Attention deficit hyperactivity disorder; OCD, Obsessive compulsive disorder.

### 3.4 Prenatal Tobacco Exposure Associated With Differences in Network Connectivity

A linear regression was performed to assess the significance of each of the five prenatally exposed substances on network‐to‐network connectivity. With PTE, there was decreased intra‐network connectivity within the default mode network and higher resting state inter‐network connectivity between the default mode and dorsal attention networks when compared to adolescents without prenatal substance exposure (Table 5). Prenatal exposure to alcohol, marijuana, cocaine, or opioids was not significantly associated with differences in network connectivity.

**TABLE 5:** The significance of demographic characteristics and prenatal substance exposure on resting‐state network correlation was assessed using a general linear model (*n* = 6674). Between and within network correlations for which prenatal tobacco exposure was a significant predictor are shown in this table.

Network 1	Network 2	*t*‐stat	*p*‐value	FDR
Default mode	Dorsal attention	3.31	9.32E‐04	0.023
Dorsal attention	Dorsal attention	−3.38	7.35E‐04	0.023

A false discovery rate (FDR) < 0.05 was considered significant.

### 3.5 Altered Functional Network Connectivity Associated With Behavioral Scale Scores

A linear regression was performed to assess the significance of network connectivity on behavioral scores. Demographic variables that were significantly associated with behavioral scores on prior analysis, namely, sex, PTE, PME, food insecurity, household income, and race, as well as scanner manufacturer, were included in the model. There were significant differences in intra‐ and inter‐network correlations with five behavioral scale scores: depression, sluggish cognitive tempo, conduct disorder, OCD, and ADHD (Table 6). Overall, there was increased inter‐network connectivity and lower intra‐network connectivity associated with higher scores on the behavioral scales with few exceptions. Alterations in default mode and dorsal attention network (DAN) connectivity were the most frequent, but ventral attention, salience, auditory and visual network connectivity also showed significant associations with behavioral scale scores.

**TABLE 6:** Significant intra‐ and inter‐network correlations of resting state networks on behavioral scores using a general linear model (*n* = 6674) and corrected for significant sociodemographic variables including prenatal substance exposure.

Behavioral Scale	Network 1	Network 2	*t*‐stat	*p*‐value	FDR
**Depression**	**Inter‐network**				
	Auditory	Salience	2.82	0.005	0.042
	Default mode	Dorsal attention	3.73	< 0.001	0.010
	Dorsal attention	Default mode	2.8	0.005	0.042
	Salience	Auditory	3.17	0.002	0.038
	**Intra‐network**				
	Default mode	Default mode	−2.82	0.005	0.042
	Dorsal attention	Dorsal attention	−2.92	0.004	0.042
**Sluggish Cognitive Tempo**	**Inter‐network**				
	Default mode	Dorsal attention	5.82	< 0.001	< 0.001
	Dorsal attention	Default mode	4.69	< 0.001	< 0.001
	**Intra‐network**				
	Default mode	Default mode	−5.39	< 0.001	< 0.001
	Dorsal attention	Dorsal attention	−4.23	< 0.001	< 0.001
	Auditory	Auditory	−2.86	0.004	0.042
**Conduct Disorder**	**Inter network**				
	Default mode	Dorsal attention	4.14	< 0.001	0.002
	Dorsal attention	Default mode	3.35	< 0.001	0.013
	Ventral attention	Frontoparietal	2.82	0.005	0.04
	Visual	Auditory	2.89	0.004	0.038
	**Intra‐network**				
	Dorsal attention	Dorsal attention	−3.71	< 0.001	0.005
	Salience	Salience	3.15	0.002	0.02
**Obsessive Compulsive Disorder**	**Inter‐network**				
	Default mode	Dorsal attention	4.01	< 0.001	0.005
	**Intra‐network**				
	Dorsal attention	Dorsal attention	3.13	0.002	0.035
**Attention‐Deficit Hyperactivity Disorder**	**Inter‐network**				
	Auditory	Ventral attention	3.19	0.001	0.007
	Default mode	Dorsal attention	5.55	< 0.001	< 0.001
	Default mode	Ventral attention	−3.56	< 0.001	0.002
	Dorsal attention	Default mode	4.42	< 0.001	< 0.001
	Dorsal attention	Ventral attention	3.76	< 0.001	0.002
	Frontoparietal	Ventral attention	2.57	0.01	0.039
	Salience	Auditory	2.67	0.008	0.031
	Ventral attention	Auditory	3.22	0.001	0.007
	Ventral attention	Default mode	−3.65	< 0.001	0.002
	Ventral attention	Dorsal attention	3.57	< 0.001	0.002
	Ventral attention	Frontoparietal	2.78	0.006	0.025
	**Intra‐network**				
	Default mode	Default mode	−4.31	< 0.001	< 0.001
	Dorsal attention	Dorsal attention	−4.59	< 0.001	< 0.001

A false discovery rate (FDR) < 0.05 was considered significant.

### 3.6 Altered Functional Network Connectivity Associated With Behavioral Scale Scores in Substance‐Exposed Subgroups

A linear regression was performed to assess the significance of network connectivity on behavioral scores within subgroups of substance exposure. Demographic variables that were significantly associated with behavioral scores on prior analysis, namely, sex, food insecurity, household income, and race, as well as scanner manufacturer, were included in the model. Prenatal exposure to tobacco, marijuana, and combined exposure was assessed. Overall, there were 205 subjects with PTE only, 55 subjects with PME only, and 26 subjects with PTE and PME only. In those with PTE exclusively, somatic problems were significantly associated with greater connectivity between the auditory and visual networks. Additionally, SCT was significantly associated with less connectivity between the dorsal attention and frontoparietal networks (FPNs). In those with PME exclusively, stress was significantly associated with less connectivity between the default mode and ventral attention networks (VANs). There were no significant alterations in network connectivity in those with PME and PTE combined. The data is summarized in Table 7.

**TABLE 7:** Significant associations between network connectivity and behavioral scores with subgroups for prenatal tobacco exposure (*n* = 205) and prenatal marijuana exposure (*n* = 55). This was based on a linear model correcting for demographic variables.

Substance	Behavioral Scale	Network 1	Network 2	*t*‐stat	*p*‐value	FDR
Tobacco	Somatic problems	Auditory	Visual	3.33	0.001	0.038
		Visual	Auditory	3.21	0.002	0.038
	Sluggish cognitive tempo	Dorsal attention	Frontoparietal	−3.36	0.001	0.023
		Frontoparietal	Dorsal attention	−3.36	0.001	0.023
Marijuana	Stress	Default mode	Ventral attention	−3.42	0.001	0.035
		Ventral attention	Default mode	−3.42	0.001	0.035

A false discovery rate (FDR) < 0.05 was considered significant.

## 4 Discussion

In this study, we showed that CBCL Scale scores from a large adolescent cohort of 6674 subjects from the ABCD database correlated with prenatal substance exposure, sociodemographic variables, and brain resting‐state functional network connectivity. The CBCL is typically used as a general screening tool whenever there is a suspicion of an emotional or behavioral problem with a child 6–18 years of age and has been validated in several studies. Of the sociodemographic variables, sex, race, annual household income, and food insecurity were associated with multiple CBCL scale scores. Male sex was associated with higher scores for most behavioral scales, except for somatic problems, where being female was linked to higher scores. Compared to Whites, Black adolescents were significantly associated with lower scores (improved outcomes) for stress, anxiety, depression, somatic problems, OCD, conduct disorder, and oppositional defiant disorder. Children of other or mixed races were also associated with lower oppositional defiant disorder scores.

Unsurprisingly, higher annual household income tended to be associated with lower (better) scores on multiple CBCL scales. Conversely, the presence of food insecurity was a strong predictor of poor behavioral scores. Hence, socioeconomic adversities are an important factor that needs to be considered in the evaluation of behavioral outcomes in prenatal substance exposures. Prior studies have demonstrated that children brought up in situations of lower income or socioeconomic status are more likely to develop ADHD (Barkley 2012). Additionally, children brought up in situations involving food insecurity are also more likely to develop and exacerbate mental health and neurodevelopmental conditions (Lu et al. 2019). On the other hand, the presence of a maternal college degree did not impact behavioral scores in this study. Previous studies have shown that maternal education impacted child cognition and development (Jackson et al. 2017; Vikram and Vanneman 2020). Since the dependent variable here is a measure of behavior rather than neurocognition, it was likely not associated with maternal education. Taken together, in addition to prenatal substance exposure, some socioeconomic factors play a pivotal role in the risk of development of mental health issues.

Of the five common prenatal substance exposures that we studied—tobacco, alcohol, marijuana, cocaine, and opioids—we identified CBCL behavioral scale scores to be correlated with prenatal tobacco exposure (PTE) and prenatal marijuana exposure (PME). Similar to the results from our study, there have been other studies that show that PTE and PME are linked to an increased risk of developing ADHD (He et al. 2020; Roncero et al. 2020). In our study, prenatal exposure to alcohol, cocaine, or opioids was not linked to alterations in behavioral outcomes for any of the CBCL‐derived scales when accounting for sociodemographic variables. However, a link between prenatal alcohol exposure and ADHD has been previously reported (Han et al. 2015). Additionally, links between ADHD and prenatal exposure to cocaine and opioids have also been previously reported (Morrow et al. 2009; Tronnes et al. 2021). Since the number of preadolescent children exposed to these substances in this dataset was relatively small, and given the strong correlations of socioeconomic adversity with behavioral scores, it is possible that the small effects of exposure to these substances were overshadowed.

When evaluating the association of prenatal substance exposure to alterations to functional brain network connectivity, only PTE was associated with differences in brain network connectivity after multiple comparison correction. Adolescents with PTE had higher inter‐network connectivity between the DMN and DAN and lower intra‐network connectivity within the DAN. 5 of the 9 CBCL scale scores, namely depression, SCT, conduct disorder, obsessive compulsive disorder (OCD), and ADHD, were associated with altered intra‐ and inter‐network brain functional connectivity, predominantly involving the DMN and DAN. For all these scales, there was significantly higher inter‐network connectivity between the DMN and DAN and significantly lower connectivity within the DAN and DMN except for OCD, where there was higher intra‐network connectivity within the DAN.

Depression was also linked with increased connectivity between the salience and auditory networks. Conduct disorder was additionally linked with greater connectivity within the SN, greater connectivity between the VAN and FPN, and greater connectivity between the visual and auditory networks. Greater SCT scores were also associated with reduced connectivity within the auditory network. Additionally, for ADHD, higher scores were associated with decreased connectivity between the DMN and VAN and increased connectivity between the DAN and VAN. Other notable associations include increased connectivity between the auditory network and VAN, increased connectivity between the FPN and VAN, and increased connectivity between the SN and auditory network.

Each of the networks mentioned has specific functions that may play important roles in behavioral symptoms. Increased connectivity between the DMN and DAN has been linked to greater self‐reported depression, and functional connectivity between these two networks mediated sleep duration (Xu et al. 2023). DMN activation has been linked to rumination, which is strongly linked to depression (Zhou et al. 2020). Male adolescents with conduct disorder have shown altered DMN connectivity (J. Zhou et al. 2016). Additionally, antisocial behavior has been linked with reduced efficiency in the FPN (Tillem et al. 2023). Reduced connectivity (Broulidakis et al. 2022) and decreased network homogeneity (Uddin et al. 2008) in the DMN have been associated with ADHD. Additionally, alterations in DMN connectivity have been linked to differences in response inhibition (dos Santos Siqueira et al. 2014; van Rooij et al. 2015), social dysfunction (Fateh et al. 2023), and temporal discounting (Broulidakis et al. 2022). In those with adult ADHD, lower resting state connectivity in the dorsal and ventral attention networks has been linked with increased symptom severity (McCarthy et al. 2013). With hypoconnectivity of the DAN being associated with ADHD, the association of reduced connectivity within this network with PTE is noteworthy.

Other networks have also been shown to be altered in ADHD. Altered connectivity between the SN and VAN has been linked with tobacco use and ADHD (Janes et al. 2018). Altered connectivity in those with ADHD has also been observed between the SN, DMN (Gonzalez‐Madruga et al. 2022; Sutcubasi et al. 2020) and executive control network (von Rhein et al. 2017). Asymmetry in visual networks have previously been linked with ADHD (Hale et al. 2014). This is also important since ADHD is linked with poorer visual processing (Kibby et al. 2015; Redondo et al. 2019), language processing (Purvis and Tannock 1997), and motor function (Dahan and Reiner 2017; Mokobane et al. 2019).

We identified PME and PTE, as well as sociodemographic variables of family income, race, and food insecurity, as being associated with adolescence behavioral scores. Considering that those with prenatal substance can be linked to negative developmental outcomes, alterations in these networks may also be important to identifying those who may be at greater risk for behavioral and neurodevelopmental disorders. Future studies need to further investigate the potential impact of prenatal substance exposures on brain regions involved with attention. The results of our study emphasize the importance of considering a biopsychosocial model in considering such implications of prenatal substance exposure on adolescent behavior, and hence on management strategies.

A few limitations need to be addressed. Even though the size of the ABCD database is almost 12,000 subjects, and 6674 subjects were used in our analysis, there are a relatively few children who were prenatally exposed to certain substances—particularly in the case of prenatal exposure to cocaine and opioids. While the presence of such a large dataset allows for assessing smaller effect sizes of some of the variables, such as prenatal substance exposure, we expect some recall bias as data was collected retrospectively. Although network correlation metrics were not identical in both directions for a few of the samples, probably related to effects of other network interactions, there was similar directionality to these network interactions. Despite these limitations, our study has shown the importance of socioeconomic adversities on behavioral scores, especially in the setting of prenatal substance exposures and their correlations with brain functional networks.

## 5 Conclusion

This study investigated potential differences in resting‐state network connectivity based on prenatal substance exposure and their impact on behavioral scores using a subset of the ABCD study database. Prenatal exposure to tobacco and marijuana was associated with worse behavioral scores. Additionally, income and food security were also significant factors for more severe behavioral outcomes. Prenatal tobacco exposure was the only substance that had a significant association with differences in functional connectivity in resting‐state networks. Five CBCL scales were associated with changes in functional connectivity—depression, sluggish cognitive tempo, conduct disorder, OCD, and ADHD. Taken together, PTE may impact brain resting‐state networks, which are linked to behavioral and neurodevelopmental conditions. Future work will need to address the overall impact of prenatal substance exposure on brain networks and test if these differences could be an effective indicator for the risk of neurodevelopmental disorders.

## Author Contributions

R. V. contributed to conception, design, analysis, and manuscript preparation. Y. Z. contributed to analysis and manuscript preparation. R. R. contributed to funding, conception, design, analysis, and manuscript preparation.

## Funding

This work was supported by Grant 2021258 from the Doris Duke Charitable Foundation through the COVID‐19 Fund to Retain Clinical Scientists collaborative grant program to RR and was made possible through the support of Grant 62288 from the John Templeton Foundation. The opinions expressed in this publication are those of the author(s) and do not necessarily reflect the view of the John Templeton Foundation.

## Conflicts of Interest

The authors have conducted this research in the absence of any commercial, financial, or personal relationships that would be considered a conflict of interest.

## Data Availability

Data used in the preparation of this article were obtained from the Adolescent Brain Cognitive Development^SM^ (ABCD) Study (https://abcdstudy.org), held in the NIMH Data Archive (NDA). The study in this article was not preregistered.
